# Clinical manifestations of pain in patients suffering from COVID-19 infected with Delta variant of SARS-Cov-2

**DOI:** 10.3389/fpain.2023.1282527

**Published:** 2023-11-15

**Authors:** Ali Mohammadian Erdi, Minoo Zakavi, Mohammad Amani, Shahnaz Fooladi, Ali Abedi

**Affiliations:** ^1^ Department of Anesthesiology, School of Medicine, Adabil University of Medical Scienes, Ardabil, Iran; ^2^ Department of Physiology, School of Medicine, Ardabil University of Medical Sciences, Ardabil, Iran

**Keywords:** pain, myalgia, arthralgia, localization, severity, COVID-19

## Abstract

**Background:**

Although respiratory presentations of COVID-19 predominate, the extra pulmonary involvement such as muscle pain, joint pain, headache, back pain, abdominal pain, and sore throat are usually included in the clinical picture of the disease and it can be considered as an early symptom in COVID-19 patients. The objective of the present study was to determine the frequency, localization, and intensity of pain in COVID-19 patients hospitalized in Imam Khomeini hospital of Ardabil, Iran.

**Methods and materials:**

A prospective study was conducted on 388 COVID-19 patients who were admitted to Ardabil, Iran's Imam Khomeini Central Hospital between March and June 2020. Demographic characteristics of patients and general clinical manifestations of pain at the first admission to the hospital, localization, severity, and continuity of pain were evaluated by using a questionnaire and the Visual Analog Scale (VAS).

**Findings:**

For the 388 (51.3% female, age 47.25 + 15.55 and 48.7% male, age 50.12 + 15.26 years old) Delta COVID-19 patients, the median duration from illness onset to hospitalization was 5 days. Patients' complaints included 89.7% fatigue, 85.56% cough, 67.8% fever, 64.17% loss of taste, 63.91% loss of smell, 37.9% diarrhea, and 11.85% skin lesions, respectively. Pain including muscle, joint, bone and low back pain was the chief complaint in both sexes. Pain complaints had started on average 5 days before admission. The distribution of pain was 313 (80.41%) muscle pain, 264 (70.61%) joint pain, 299 (77.1%) headache, 208 (53.6%) low back pain, 312 (80.41%) sore throat, and 157 (40.46%) abdominal pain. Out of 388 patients, 292 (75.25%) had diffuse pain.

**Conclusions:**

Acute pain including myalgia, sore throat, arthralgia, headache, and low back pain were the most common symptoms of COVID-19 patients. Viral diseases such as COVID-19 may trigger the immune system to release cytokines that lead to muscle pain. Patients presenting to healthcare centers with complaints of pain should be evaluated for suspected COVID-19 infection.

## Introduction

The coronavirus known as SARS-CoV-2, which caused severe acute respiratory syndrome, is the cause of the ongoing COVID-19 pandemic (1, 2).

In December 2019, an outbreak in the Chinese city of Wuhan led to the discovery of the novel virus (3). There were failed attempts to contain it, which allowed the virus to become widespread throughout Asia and eventually the entire world. The World Health Organization (WHO) declared the outbreak a pandemic and a public health emergency of international concern on January and March, 2020 (4). By March 20, 2022, more than 468 million COVID-19 cases reported, with a fatality rate of 1.3% and the pandemic was one of the deadliest in history as of January 2023, with more than 670 million cases and 6.7 million confirmed deaths (4).

Since then, the world has been shaken by a series of COVID-19 outbreak waves brought on by mutant strains of the pathogens like the severe acute respiratory syndrome coronavirus 2 (SARS-CoV-2) (5, 6). The variants of SARS-CoV-2 including alpha, beta, gamma, delta, and omicron have all been identified as variants of concern (VOCs) due to their high virulence and infectivity (7).

The dominant circulating strain of SARS-CoV-2 that is currently sweeping the globe is the omicron variant (8). The predominant omicron sublineages found in China's regional COVID-19 outbreaks are BA.1 and BA.2 (9). The virus was first identified in South Africa in Nov 2021, that the infection was linked with markedly shorter hospital stays, lower severity and mortality compared to earlier COVID-19 hits (10).

The viral genome of the omicron variant, as opposed to those of the other VOCs, had significantly more mutations (11).

The severe acute respiratory syndrome coronavirus type 2 (SARS-CoV-2) can have an impact on almost all organ systems, resulting in symptoms in the respiratory, musculoskeletal, cardiovascular, and neurologic systems (12–14). In previous epidemics, nociplastic pain, a new type of pain for which central sensitization is thought to be largely responsible, has also increased (15).

In addition to the well-known ability of viral illnesses to cause muscle pain and headaches, pathogens like COVID-19 may also cause pain through psychological stress, deconditioning brought on by quarantine, and other mechanisms (16).

The most frequent symptoms of COVID-19 are fatigue, dry cough, and fever, which can range from being undetectable to being fatal. Only a small number of the more than 50,000 peer-reviewed articles on COVID-19 infection that have been released since January 2020 focused on how the disease affects pain, the majority, instead, concentrated on logistical and educational issues (17, 18).

The respiratory, musculoskeletal, cardiovascular, neurological, and gastrointestinal symptoms caused by Coronavirus 2 (SARS-CoV-2) first appeared in 2019 and spread to nearly every organ system (13, 19).

Although earlier epidemics have also led to an increase in pain, a new category in which central sensitization is thought to play a significant role, has emerged (16).

One of the most significant and frequent causes of admission to the hospital is pain (20). The COVID-19 infection pandemic has had pain as one of its primary symptoms since 2019. it has a wide clinical spectrum, ranging from asymptomatic to clinical conditions that may cause multiple organ failures. The most frequent pain symptoms in COVID-19 are myalgia and headache, but sore throat, abdominal pain, and chest pain also present (21). Myalgia is the most prevalent musculoskeletal symptom among COVID-19 patients, with prevalence rates ranging from 30 to 36 percent (22, 23). There were a surprising number of COVID-19-positive patients who presented with pain but no initial respiratory tract symptoms. As a result, pain may be a symptom of COVID-19 infection (17). We proposed the possibility that pain could be a symptom for virulence and be linked to worse outcomes. The primary main objectives of this study were to identify and assess the frequency, localization, and severity of pain among the COVID-19 patient population's presenting signs and symptoms.

## Materials and methods

A prospective, descriptive and analytical study included 388 patients with a definite diagnosis COVID-19 who admitted to the Imam Khomeini central Hospital of Ardabil (Ardabil University of Medical Sciences), IRAN, from March to June 2022. All positive COVID-19 patients diagnosed by PCR testing who admitted to hospital studied. Exclusion criteria were patients with diabetic and malignancy, pregnancy, blood disease, autoimmune deficiency, and patients who died in the time of study, and patients who were younger than 15 years old. The study approved by the Research Ethics Commission of Arums with approval code IR.ARUMS.REC.1400.064. All patient information remained confidential and written consent obtained from the patients.

The study protocol was conducted by a questionnaire including demographic characteristic such as sex, age, height and weight, medication, additional disease, presence of pre-existing concomitant pain conditions, and clinical manifestations of pain pertaining to the location of pain. Pain score on a Visual Analog Scale (VAS) of 0 to 10 recorded at the time of admission and before analgesic interventions (24).

## Statistical analysis

Data was collected and IBM SPSS ver. 25 program was used for statistical analysis. Descriptive data were expressed as mean ± SD or median (IQR) for continues variables and frequency was applied for nominal data. Demographic characteristics and clinical symptoms are expressed in percentages and number of participants. The normal distribution was evaluated using the Kolmogorov-Smirnov test.

The chi-square and ANOVA tests were used to compare categorical variables and examine the correlation between variables, respectively. Regression model was used to determine the correlation between age and pain score and the normality test for the age and pain score was performed by the Shapiro test. Finally, the confidence level of 95% was kept and *P* value less than 0.05 was considered significant.

## Results

Demographic characteristics are summarized in Table 1. In our study, a total 388 patients (51.3% were female and 48.7% were male) with COVID-19 hospitalized in Imam Khomeini hospital of Ardabil, IRAN, from February to March 2022. The mean age for females and males were 47.25 + 15.55 (median age, 47) and 50.12 + 15.26 (median, 50) years old, respectively and there was no significant difference. The duration time from illness onset to hospitalization was 5 days. The mean weight for men and women was 80.87 ± 11.15 and 71.03 ± 13.22 kg, respectively, and the differences was statistically significant (*P* < 0.001) but there were no differences between height and BMI. Of the patients, 4.23% of men and 1.5% of women were smokers, 6.8% of men and 11.55% of women had history of covid (Tables 1, 2).

**Table 1:** Demographic characteristic, clinical symptom, in patients with COVID-19 infected by the omicron variant in Ardabil, IRAN, in February and March 2020.

Total (*n* = 388)				
Sex		Male (*n* = 189)	Female (199)	*P* value
Age (year)		50.12 ± 15.26	47.25 ± 15.55	0.067*
Height (Cm)		174.21 ± 6.9	163.11 + 5.63	<0.001*
Weight (Kg)		80.87 ± 11.15	71.03 ± 13.22	<0.001*
BMI		26.65 ± 3.54	26.77 ± 5.29	0.804*
Smoking, *n*	No (364)	168	196	<0.001**
	Yes (24)	21	3	
History of Covid, *n*	No (352)	176	176	0.085**
	Yes (36)	13	23	
Family Covid, *n*	No (147)	66	81	0.143**
	Yes (241)	123	118	

* *T*-test.

** Chi square.

**Table 2:** Distribution of BMI range in patients with COVID-19 based on gender. Data expressed as frequency. *P* < 00.1.

		BMI range				*P* value
		Underweight (<18.5)	Healthy weight (18.5–24.99)	Over weight (25–29.99)	Obese (>30)	
Sex	Female	5	77	76	41	<0.001
		83.3%	62.1%	39.2%	64.1%	
	Male	1	47	118	23	
		16.7%	37.9%	60.8%	35.9%	

Data expressed as frequency and percent.

Complaints of the patients were 78.1% dyspnea, 80.41% myalgia, 70.61% arthralgia, 76% bone pain, 75.3% diffuse pain, 40.5% abdominal pain, 80.4% sore throat, 77.1% headache 89.7% fatigue, 67.8% fever, 64.2% loss of taste, 37.9% diarrhea, 15.7% skin lesion 85.56% cough, 43.5%, 63.91 loss of smell (Table 3). The 49.8% of the patients with fever were men, and 50.2% were women and 48.2% of men and 51.8% of women had cough. Loss of smell was similar in women and men, while loss of taste sense was more common in men (50.6%) than in women (49.4%). 51.7% of women and 48.3% of had diarrhea. Skin lesion for men and female was 37.7% and 62.3%, respectively.

**Table 3:** Clinical symptoms of patients with COVID-19 infected by the Delta variant in Ardabil, IRAN, in February and March 2020.

		Total (388)		*P* value
		Male (189)	Female (199)	
Fever, *n*	No (125)	58	67	0.32
	Yes (263)	131	132	
Cough, *n*	No (56)	29	27	0.362
	Yes (332)	160	172	
Diarrhea, *n*	No (241)	118	123	0.401
	Yes (147)	71	76	
Loss of smell, *n*	No (140)	65	75	0.284
	Yes (248)	124	124	
Loss of taste, *n*	No (139)	63	76	0.186
	Yes (249)	126	123	
Skin lesion, *n*	No (327)	166	161	0.041
	Yes (61)	23	38	

Data expressed as frequency.

Pain complaints had started on average 4.18 days before admission to the hospital. 75.25% of the patients with pain had diffuse pain. Also, 55.75% of the patients with widespread pain were female. Among patients reporting pain in presentation or during the illness, the distribution of location was 312 myalgia (51.6% women), 274 arthralgia (53.64% women), 299 headache (53.84% women), 208 low back pain (57.21% women), 348 bone pain (52.87% women), 164 sore throat (52.56% women), and 157 abdominal pain (56.68% women) (Table 4). In the present study, the commonest age groups for both sexes with Covid-19 were 31 to 40 years old (Table 5). Our result showed that 194 patients (60.8% men and 39.2% women) were overweight and a statistically significant relation was found between sex and BMI (*P* < 0.001).

**Table 4:** Location distribution of pain in patients with COVID-19 infected by the Delta variant in Ardabil, IRAN, in February and march 2020.

		Sex		
		Female (199)	Male (189)	*P* value
Dyspnea, *n*	Yes (303)	157	146	0.174
	No (85)	42	43	
Low back pain, *n*	Yes (208)	119	89	.014
	No (180)	80	100	
Muscle pain, *n*	Yes (312)	161	151	0.431
	No (76)	38	38	
Joint pain, *n*	Yes (274)	147	127	0.181
	No (114)	52	62	
Bone pain, *n*	Yes (348)	184	164	0.192
	No (40)	15	25	
Diffuse pain, *n*	Yes (292)	154	138	0.388
	No (96)	45	51	
Fatigue, *n*	Yes (348)	184	164	0.069
	No (40)	15	25	
Abdominal pain, *n*	Yes (157)	89	68	0.098
	No (231)	110	121	
Sore throat, *n*	Yes (312)	164	148	0.370
	No (76)	35	41	
Headache, *n*	Yes (299)	161	138	0.071
	No (89)	38	51	

Data expressed as frequency.

**Table 5:** Distribution of age range in patients with COVID-19 based on gender. Data expressed as frequency. *P* = 0.128.

Age Group									
		<20	21–30	31–40	41–50	51–60	61–70	>71	*P* value
Sex	Male (189)	1	27	51	40	38	24	18	0.128
	Female (199)	4	14	39	39	41	36	16	

Data expressed as frequency.

While regarding the correlation between age, BMI and severity of pain, we found that in both sexes severity of pain was increasing with age.

## Association between age and disease severity

VAS score for clinical manifestation of pain has been summarized in Table 6. Among patients with pain complaints, the median VAS score for fatigue, and sore throat was 6 for both sexes while the median for muscle pain was 5 and for low back pain, Joint pain, and abdominal pain was 4 for both sex. In addition, the median VAS score of diffuse pain and headache was 5 and 4 for female and male, respectively.

**Table 6:** VAS score for clinical manifestation of pain in patients with COVID-19 infected by the Delta variant in Ardabil, IRAN, in February and march 2020.

		Score (VAS)										
		1	2	3	4	5	6	7	8	9	10	Total
Dyspnea (*n*)5	Female (Median = 4, IQR = 3–7)	9	11	28	34	17	14	9	11	2	22	157
	Male (Median = 5, IQR = 4–7)	1	6	26	36	27	11	9	6	4	20	146
Low Back Pain (*n*)4	Female (Median = 4, IQR = 3–6)	8	11	23	26	13	10	6	8	3	11	119
	Male (Median = 4, IQR = 3–6)	13	18	15	15	15	7	3	4	3	8	89
Muscle Pain (*n*)5	Female (Median = 5, IQR = 3–7)	2	11	29	27	32	16	13	8	8	20	166
	Male (Median = 5, IQR = 4–6)	3	5	24	41	26	20	10	7	4	11	151
Joint Pain (*n*)	Female (Median = 4, IQR = 3–7)	6	11	33	30	17	12	11	5	6	16	147
	Male (Median = 4, IQR = 3–6)	5	11	28	35	15	20	3	4	3	3	127
Bone Pain (*n*)	Female (Median = 4, IQR = 3–7)	6	14	29	31	23	11	10	5	12	15	156
	Male (Median = 4, IQR = 3–6)	5	14	25	35	22	14	5	6	5	7	138
Diffuse Pain (*n*)	Female (Median = 5, IQR = 3–7)	8	16	16	32	21	12	12	12	9	16	154
	Male (Median = 4, IQR = 3–7)	7	11	36	26	14	7	9	7	7	14	138
Sore Throat (*n*)	Female (Median = 6, IQR = 4–8)	7	6	9	22	30	24	21	12	19	14	164
	Male (Median = 6, IQR = 5–7)	1	6	5	19	24	42	23	10	7	11	148
Abdominal Pain (*n*)	Female (Median = 4, IQR = 3–6)	8	12	18	12	16	8	4	3	0	8	89
	Male (Median = 4, IQR = 3–5)	5	8	19	13	8	5	3	1	1	5	68
Headache (*n*)	Female (Median = 5, IQR = 3–7)	7	11	29	26	25	13	15	3	3	29	161
	Male (Median = 4, IQR = 3–6)	2	18	32	24	16	14	4	7	4	17	138
Fatigue (*n*)	Female (Median = 6, IQR = 5–9.5)	3	8	9	22	29	22	12	20	13	46	184
	Male (Median = 6, IQR = 4–7.5)	2	10	17	23	29	28	14	10	10	21	164

There was a statistically significant correlation between complain of pain and sex, fatigue, loss of taste and smell, pain intensity, and localized pain. A statistically significant correlation was found between back pain with abdominal pain and female gender, and between headache and age. A statistically significant correlation was found between the number of days between the onset of pain and the admission and the severity of pain, widespread pain, ongoing pain, and pulmonary involvement. There was also a significant association between ongoing pain and pain intensity. Statistically significant correlation analysis results are shown in Table 3, 4.

Severity of Dyspnea, bone pain, and diffuse pain increased with age in both sex. Also, low back pain, Muscle pain, headache and fatigue increase in men but decreased in women.

## Discussion

Covid-19 is a respiratory infection caused by the SARS-CoV-2 virus that usually causes flu-like symptoms, including shortness of breath, fever, cough, and fatigue. Since 2019, this weird virus has killed millions of people around the world.

Although understanding the symptoms of COVID-19 infection is crucial for early diagnosis of the condition, the symptoms of the disease are frequently vague. The symptoms of Covid-19 frequently manifest as muscle and joint pain, fever, cough, abdominal pain, or fatigue.

In some cases, patients may initially show no symptoms or only have muscle pain, headache, nausea, diarrhea, and a few days prior the fever (18).

Our results showed that patients with covid-19 presented with clinical manifestations of pain such as myalgia, arthralgia, low back pain, fatigue, and abdominal pain.

The most usual symptoms involved general symptoms such as myalgia, arthralgia, and headaches, which are common in acute viral illnesses (9).

Pain is an unpleasant sensory and emotional experience associated with actual or potential tissue damage, or described in terms of such damage.

Pain is not solely a physical sensation but also includes emotional and cognitive components. It can be caused by various factors, including injury, illness, or other noxious stimuli. The subjective nature of pain means that it is ultimately defined by the individual experiencing it, making it difficult to measure objectively but studied extensively in fields such as neuroscience, psychology, and medicine.Viral infections can cause muscle pain through various mechanisms, including cytokine activation such as IL-1, IL-6, IL-12, IFN-*γ*, and TNF-α and stimulation of adrenergic receptors found in skeletal and smooth muscle fibers (25). Viral-induced arthralgia has a similarly complex pathophysiology that can be brought on by immune complex formation, direct invasion of the joint, and immune modulation leading to chronic inflammation (19).

Headaches are caused by a variety of factors, such as trauma, vascular and myofascial pathology, and stress, all of which are frequently present in COVID-19 infection (26, 27).

In contrast to other studies, the prevalence of pain in our study was 534 percent (207 of 388) among COVID-19 patients.

In a study done on 99 covid-19 patients, the prevalence of myalgia, headache, chest pain, and abdominal pain was 11%, 8%, 2%, and 3%, respectively (28).

Another study conducted at a hospital in Wuhan on 138 patients revealed that 35% had myalgia, 7% had headaches, and 2% had abdominal pain (29).

However, common cold and flu patients experience myalgia and headaches. Additionally, up to 90% of people with Chikungunya viral infection report experiencing arthralgia and myalgia (30).

Other potential mechanisms by which viral infections may deteriorate pain include pain sensitization, stressors like depression and anxiety, sleep deprivation, and sympathetic nervous system activation (31).

pain is believed to be a symptom of pathogenicity or viral bad load, both of which may exacerbate chronic pain. Pain signals may be suppressed by a strong systemic stimulus such as anxiety and respiratory distress, which is similar to the modulation of conditioned pain. Furthermore, Increased catecholamines associated with severe respiratory distress may reduce pain signaling (32).

One study showed that myalgia and headache were the most common symptoms with a prevalence of 30%–36% and 58.33% in patients with COVID-19 (33). In some cases, there is no respiratory symptoms in covid-19 patients with diffuse pain.

Pain intensity associated with COVID-19, has been reported to be mild to moderate (17, 21). In our study, the mean pain intensity assessed by VAS was moderate and in accordance with the literature (20, 34).

A study showed that headache (13.6%), muscle and joint pain (14.9%) were the most common symptoms of patients with covid-19 (21). The prevalence of myalgia arthralgia has been reported to be higher in other studies, reaching 59 percent (35, 36).

In our study, the most common symptoms were muscle pain, joint pain, and headache with a prevalence of 80.41, 70.61% and 77.1%, respectively, which was much higher than previous reports (12, 35).

Patients with covid-19 may experience gastrointestinal symptoms like abdominal pain, nausea, vomiting, anorexia, and diarrhea. The frequency of abdominal pain in patients with covid-19 is reported to be 2 to 8% (29, 37). Compared to other studies (22, 29), our study had a higher than average incidence of abdominal pain (40.46%) and diarrhea (37.9%).

One of the most frequent hospitalization complaints among Covid-19 patients is pain. It occurs in about half of the cases and 2 days prior to hospital admission (20). Our findings showed that patients with pain complaints should be assessed for COVID- 19 infection.

Also, a significant correlation was observed between pain onset time and intensity of pain.

General pain and its intensity were also significantly correlated to persistent pain. Pain may be the main complaint and widespread. The severity, chronic and widespread of pain depends on the time of onset. The prognosis of the pain can be influenced by the presence of pain at the time of presentation and how quickly the pain starts (36).

Regarding the correlations between pain and other clinical manifestations, local and diffuse pain is more common in women than in men. Women present more often with pain and loss of taste and smell, and fatigue is more common in women.

Whether the pain becomes chronic or not, after COVID- 19 infection is unknown. In our study, it was impossible to generalize chronic pain, since the patients weren't followed up after discharge. However, Chronic pain and fatigue after SARS infection have been reported (38). The main point of this study was to exclude cases of death in hospitalization and death after discharge.

The data was collected by interview and questionnaire, so there is a possibility of a loss of validity. Although pain intensity was assessed by VAS, pain characteristics were not explored as continuous or intermittent.

In patients with pain complaints, COVID-19 infection should not be disregarded, so doctors should exercise caution in this area. More research is required to assess how pain patterns change over time, particularly in various treatments of COVID-19, as well as the influence of disease severity and characteristics on pain patterns (34).

Studies have shown that the covid-19 infection stimulates the secretion of pro-inflammatory molecules and cytokines. these stimulate the formation of prostaglandin E2 and affect the nervous system which leads to sending pain messages to the brain (39, 40).

Although pulmonary involvement is evident in patients with covid-19, musculoskeletal complications due to covid-19 have mostly been ignored in the acute period due to the greater importance of restoring cardiovascular and pulmonary stability and in the long term due to the neglect of patients (41).

the main effort of the treatment personnel is focused on primary treatments to save patients' lives and reduce the severity of the patient's involvement. but after discharge from the hospital, patients may complain of musculoskeletal symptoms (41, 42).

Muscle pain, joint pain, sore throat, abdominal pain, chest pain, and headache may even occur separately in patients or with pulmonary complications. So, pain should be considered as a primary symptom in patients with covid-19 (34, 43). Although we didn't follow up patients after discharge, studies show that 10%–20% of hospitalized patients still complain of pain after discharge.

In some patients with covid, due to the absence of pulmonary complications, treatment may be done according to pain (44).

Therefore, it is recommended to all the colleagues of the medical staff who are in the first line of patient evaluation to consider all patients as possible sufferers and to consider precautionary measures in encounters.

## Conclusion

Our findings showed that body pain, caused by the immune system's response to the covid-19 infection, can appear in the early stage of the disease. So, pain should be considered a symptom of covid, and having a PCR test and CT is necessary (45, 46). In addition, age should be considered as a risk factor in patients with covid-19 in men and women (47). Finally, performing appropriate therapeutic exercises by mechanical therapy is recommended. using breathing training devices can increase oxygen saturation of blood and leads to decrease fatigue. Also, changing the position of the patient during hospitalization and after discharge leads to decrease fatigue, muscle pain and lifestyle should be changed (48).

## Data Availability

The original contributions presented in the study are included in the article/Supplementary Material, further inquiries can be directed to the corresponding author.
